# Identification and complete genome sequencing of a divergent olive virus T isolate and an olive leaf yellowing-associated virus isolate naturally infecting olive trees in Greece

**DOI:** 10.1007/s11262-022-01934-4

**Published:** 2022-09-24

**Authors:** Asimina Katsiani, Polina Panailidou, Matthaios Mathioudakis, Nikolaos Katis, Varvara I. Maliogka

**Affiliations:** 1 Plant Pathology Laboratory, Faculty of Agriculture, Forestry and Natural Environment, School of Agriculture, Aristotle University of Thessaloniki, 54124 Thessaloniki, Greece; 2 Plant Pathology Laboratory, Institute of Olive Tree, Subtropical Crops and Viticulture (IOSV), ELGO-DIMITRA, Karamanlis Ave. 167, 73134 Crete, Chania Greece

**Keywords:** Olive virus T, Tepovirus, Olive leaf yellowing-associated virus, Closteroviridae, HTS

## Abstract

Several new full genome sequences of olive viruses came to light recently via high-throughput sequencing (HTS) analysis. In this study, total RNA HTS analysis of two Greek olive trees revealed the presence of an olive virus T (OlVT) isolate and an olive leaf yellowing-associated virus (OLYaV) isolate. The full viral genome of OlVT isolate (50Ch) is composed of 6862 nucleotides encoding for three proteins (replicase, movement protein, and capsid protein) with typical betaflexiviruses’ genomic features. However, both sequence and phylogenetic data analysis exhibited high levels of variability between 50Ch and the previously characterized OlVT isolates. In addition, the almost full genome of the Greek OLYaV isolate (OL2) was obtained, which is composed of 16,693 nucleotides encoding for 11 open reading frames (ORFs) and shares common genomic features with the recently characterized OLYaV isolates from Spain and Brazil. Sequence and phylogenetic analysis revealed high similarity between these three isolates. Due to problems encountered with the detection of both viruses, new nested RT-PCR assays were developed and applied. In addition, recombination events were observed in OlVT isolates (50Ch GR-168), thus highlighting the potential role of this mechanism in the evolution of the virus. This study is adding further knowledge to the limited information available about these recently characterized olive infecting viral pathogens and highlights their widespread distribution in Greece, one of the most important olive producing countries of the world.

**Supplementary Information:**

The online version contains supplementary material available at 10.1007/s11262-022-01934-4.

## Introduction

Olive (*Olea europaea* L.) is one of the most important crops in the Mediterranean area, where it has been grown for centuries. Nevertheless, due to its vegetative propagation through cuttings, it has accumulated several pathogens, including viruses. More than seventeen viruses belonging to different genera or families have been reported to naturally infect olive trees [1–6]. Over the last decade, the wide application of high-throughput sequencing (HTS) has allowed the rapid identification and characterization of several novel or new strains of known viruses in various plant species using different technologies [7–10]. Hence, in olive, new viral agents have also come up or divergent variants of known viral agents have been molecularly characterized with the application of HTS [5, 6, 11, 12].

Olive virus T (OlVT) is a new viral species assigned to the genus *Tepovirus* (*Betaflexiviridae*), members of which have positive-sense, single-stranded (ss) RNA genomes that encode for a replication-associated protein (REP), a putative movement protein (MP), and a capsid protein (CP) [13]. So far, no vector has been reported for tepoviruses, therefore they are putatively distributed worldwide through the transportation of infected plant material. Nevertheless, potato virus T (PVT), another tepovirus, has been reported to be pollen- and seed-borne [14]. OlVT was recently found by HTS during field surveys in Central and Northern Greece, and its incidence in 158 samples tested was low (4.4%). The virus was more often found in Central Greece [5]. So far, no correlation has been reported between virus presence and symptoms development either in the field-collected samples or by grafting three olive varieties and infections were latent [5].

On the other hand, olive leaf yellowing-associated virus (OLYaV) represents a putative closterovirus detected in olive more than 20 years ago [15]. However, it remains unclassified and is not yet an approved member of the family *Closteroviridae* due to the lack of sufficient biological and molecular data [16]. *Closteroviridae* members have among the largest genomes of plant viruses composed of one up to three single-stranded (ss) positive-sense RNA segments [16]. The complex ORF1a-ORF1b encodes the replication-related proteins i.e., methyltransferase (MTR), helicase (Hel), and RNA-directed RNA polymerase (RdRp). Downstream ORFs form a five-gene module which is conserved, with few modifications, among most members of the family (6 kDa small hydrophobic protein, an HSP70h, a ~ 60 kDa protein, a CP, and a CPm) [17, 18]. Recently, the complete genome of two OLYaV isolates originating from Brazil and Spain has been determined by HTS [11, 12]. Based on the novel sequences and phylogenetic data, OLYaV was proposed to be classified as a member of a new genus within the family *Closteroviridae* along with actinidia virus 1 (AV1) and persimmon virus B (PVB) [11]. The virus has been previously reported to occur in Greece [4, 5]; however, only small genome sequences (339–383 bp from HSP70h) from four Greek isolates (GR80OL, 8Gaidourelia, 22Conservolia, and OLYaV-Gr/Ms, respectively) have been published or deposited to GenBank so far [4, 5, 18].

The aim of the present work was to study the virome of major olive varieties in Greece and unveil the presence of putative new viruses or divergent viral strains. Olive samples were collected from 85 trees, 56 of cv. Chondrolia Chalkidikis from Central Macedonia, and 4 of cv. Conservolia from a germplasm collection olive grove located in Chania, Crete. HTS of two randomly selected samples (one from Central Macedonia and one from Crete) allowed the identification of two viruses, OlVT and OLYaV, for which genomic sequence information is so far limited. Moreover, specific RT-PCR assays developed herein, using the available along with the new determined sequences, and applied in testing the collected samples have shown that both viruses are probably more frequent in Greek olive trees than previously reported.

## Materials and methods

### Olive plant material and HTS analysis

During virus surveys in olive groves taking place mainly in Northern Greece and in Crete in 2019, samples 50Ch of the cv. Chondrolia Chalkidikis (10 years old) and OL2 of the cv. Conservolia (> 40 years old), representing two of the most important Greek varieties, collected from Chalkidiki and the National Olive Repository located in Chania, Crete, respectively, were randomly selected among other 55 samples for HTS analysis. Total RNA was extracted from leaves exhibiting slightly chlorotic symptoms, with the Plant/Fungi Total RNA Purification Kit (Norgen Biotek Corporation, Thorold, ON, Canada) according to the manufacturer’s protocol and was subjected to rRNA depletion (RiboZero Plant treatment), library construction (TruSeq Stranded Total RNA), and HTS (100 bp paired-end reads) in a NovaSeq platform (Illumina, San Diego, CA, USA) at Macrogen Inc. (Seoul, S. Korea). Library construction as well as rRNA depletion were performed by Macrogen Inc (Seoul, S. Korea). The obtained reads were de novo assembled using Geneious Prime® v. 2020. 2.4 (https://www.geneious.com/). For the total RNA reads prior to de novo assembly, the host genome was removed using the same software and the quality of the resulting fastq files was controlled by running FastQC [19].

### 5′ and 3′ RACE assays and partial confirmation of the new OlVT and OLYaV isolates with Sanger sequencing

The presence of the OlVT sequence in the original tree was confirmed with three different primer pairs and Sanger sequencing of the PCR products (Suppl. Table 1).

In addition, the presence of the OLYaV sequence in the sample submitted to HTS analysis was confirmed by amplifying part of the HSP70h gene of the virus, using the primers designed for RT-PCR detection and the amplified segment was subjected to Sanger sequencing (Suppl. Table 1).

For the confirmation of the 5′ and 3′ ends of both sequences, 5′ and 3′ RACE assays were performed following the protocols of Alves-Freitas et al. [20].

### Sequence comparisons, phylogenetic reconstructions, and recombination analysis

Amino acid (aa) sequence comparisons of isolate 50Ch were performed for each ORF using available sequences of all *Tepovirus* members from the EMBL-EBI and GenBank databases. Global alignments were constructed using Muscle (MUltiple Sequence Comparison by Log-Expectation) [21]. ORFs and the identity scores in aa and nucleotides (nt) were determined with the Geneious Prime® v. 2020. 2.4 (Biomatters Ltd.).

To study the phylogenetic relationships of 50Ch sequence within the genus *Tepovirus*, a Maximum-likelihood (ML) phylogenetic tree was constructed using the complete nt sequences of 50Ch and the tepoviruses isolates available in the GenBank database. Phylogenetic trees of OlVT isolates were constructed using either full genomes or the partial replicase and CP sequences with the ML algorithm in MEGA v. 7.1 (Molecular Evolutionary Genetics Analysis) [22] using the most suitable substitution model and 1000 bootstrap replicates. For OLYaV, the complete nt sequences of isolates V64 and CS1 were used, and the tree was constructed with the ML algorithm in MEGA v. 7.1 using the GTR + I model and 1000 bootstrap replicates.

Putative recombination events between OlVT (including 50Ch) and PrVT isolates were investigated with RDP5 software, using the RDP, GENECONV, BOOTSCAN, MAXCHI, CHIMERA, SISCAN, and 3SEQ methods [23].

### Field surveys for the presence of OlVT and OLYaV

An RT-PCR assay was developed to study the spread of the OlVT isolate using the following primer pairs amplifying 353 bps and 662 bps of the viral ORFs REP: OlVTF1/OlVTR1; and CP: OlVT_CPAn_F/OlVT_CPAn_R, respectively (Suppl. Table 1). In both cases, the RT reaction took place by mixing 3 μl of total RNA of each sample with 40 U M‐MLV (GeneON), 1 μM of OligodT (18mer) primer (5′-TTTTTTTTTTTTTTTTTT-3′), 0.25 mM of each dNTP, 1X standard buffer [50 mM Tris–HCl (pH 8.3), 75 mM KCl, 3 mM MgCl_2_], and nuclease-free water up to the final volume of 20 μl and following the thermal profile of 42 °C for 60 min and 70 °C for 15 min. Subsequently, 2 μl of cDNA was used for PCR, in a new 0.2-ml tube together with 1.5 U GRS Hotstart DNA Polymerase (GRiSP), 0.2 mM of each dNTP, 1X buffer [10 mM Tris–HCl (pH 8.3), 50 mM KCl, 1.5 mM MgCl_2_], 0.4 μM of primers OlVTF1/OlVTR1 (in the case of REP reaction) or 0.25 μM of primers OlVT_CPAn_F/OlVT_CPAn_R (in the case of CP reaction) (Suppl. Table 1), and nuclease-free water till the final volume of 20 μl. PCR thermocycling conditions were the following: for REP 95 °C for 5 min, followed by 40 cycles of 95 °C for 30 s, 59 °C for 30 s, 72 °C for 30 s, and finally 72 °C for 2 min, for CP 95 °C for 5 min, followed by 5 cycles of 95 °C for 30 s, 45 °C for 30 s, 72 °C for 30 s, 35 cycles of 95 °C for 30 s, 50 °C for 30 s, 72 °C for 30 s, and a final step of 72 °C for 2 min. Overall, 85 samples collected from Central Macedonia (Suppl. Table 2) were submitted to total RNA extraction following the protocol described by Chatzinasiou et al. [24] and further tested using the previously mentioned detection methods.

Due to discrepancies in OLYaV detection with the available RT-PCR methods, a new nested RT-PCR, targeting the conserved part of the HSP70h that has been used in previous closterovirus generic assays [15, 25], was developed and applied. For primers’ design, the HTS sequences recovered in this study were used along with sequences available in the database. The new primer pair OLYaV_F_1905 (5′- TRATGTGGCCTGAACTACGTTT-3′) and OLYaV_R_2811 (5′-TCTTATAGCTTCATCAACRTC-3′) amplifies 909 bps of the viral genome, followed by a nested PCR with the primer pair OLYaV_nest_F (5′-ACWTTGTGYGTACTTGTTTCTA-3′) and dHSP do2 [25] which amplifies 781 bps of the HSP70h gene. More specifically, a one-step RT-PCR assay was carried out in which 3 μl of total RNA of each sample was added in a mixture of 25 μl final volume together with 3 U M‐MLV (GeneON), 1.5 U Taq DNA Polymerase (GenScript), 1X buffer [10 mM Tris–HCl (pH 8.3), 50 mM KCl, 1.5 mM MgCl_2_], 10 mM DTT, 0.2 mM of each dNTP, 0.8 μM of primers OLYaV_F_1905/OLYaV_R_2811, and nuclease-free water till the final volume. The RT-PCR thermocycling program was the following: 42^ο^C for 50 min, 94 °C for 3 min, 40 repetition of 95 °C for 30 s, 60 °C for 15 s, 52 °C for 15 s, 72 °C for 50 s, and a last step of 72 °C for 5 min. In nested PCR assay, a 20 μl reaction was prepared containing 1.5 U Taq DNA Polymerase (GenScript), 1X buffer [10 mM Tris–HCl (pH 8.3), 50 mM KCl, 1.5 mM MgCl_2_], 0.2 mM of each dNTP, 1 μM of each primer (OLYaV_nest_F/dHSP do2), 1μL of RT-PCR product, and nuclease-free water till the final volume and took place following a thermocycling profile of 94 °C for 3 min, 40 cycles of 95 °C for 30 s, 60 °C for 15 s, 55 °C for 15 s, 72 °C for 30 s, and finally 72 °C for 5 min. All samples collected from Central Macedonia were also tested for the presence of OLYaV (Suppl. Table 2), following the same preparation as described above for OlVT.

## Results

### HTS analysis of olive samples

The run after quality control yielded ca. 34.4 million and 35.5 million of 100-nt paired-end reads for the samples 50Ch and OL2, respectively. The 10,476 and 5535 contigs produced, respectively, from the de novo assembly of the reads using the Geneious Prime 2019.2.3 and Velvet softwares were checked for similarity against the nt/aa databases using the available online BLAST tools.

**Fig. 1:**
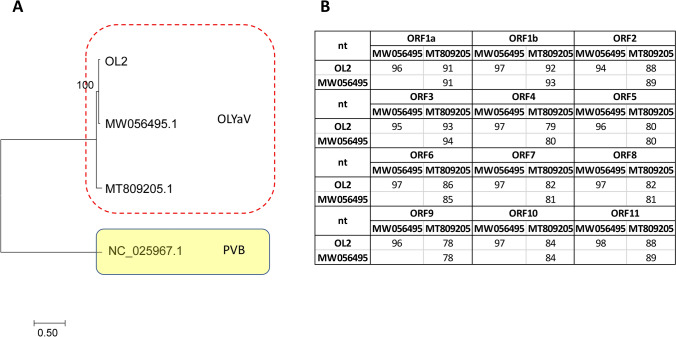
**Α** Maximum-likelihood phylogenetic tree constructed in MEGA v 7.1 using the full genome from OLYaV and **Β** similarities in nucleotide level (%) for each ORF of the isolate OL2 with the two available full sequences. The viral sequences are reported with their accession number. The scale bar shows the number of substitutions per site. Bootstrap percentages (1000 re-samples) are indicated on the branches. PVB: persimmon virus B

For sample OL2, one contig (16,693 in length) exhibited high percentage of nt sequence identity with OLYaV. The nearly complete genome (missing only 30nts from the 3′ noncoding end) (accession no. OK569886) of the OL2 closterovirus was 96.37% identical to isolate V64 (accession no. MW056495) from Spain and 87.77% to isolate CS1 (accession no. MT809205) from Brazil. These findings were also supported by the phylogenetic analysis of the full genome sequences (Fig. 1).

**Fig. 2:**
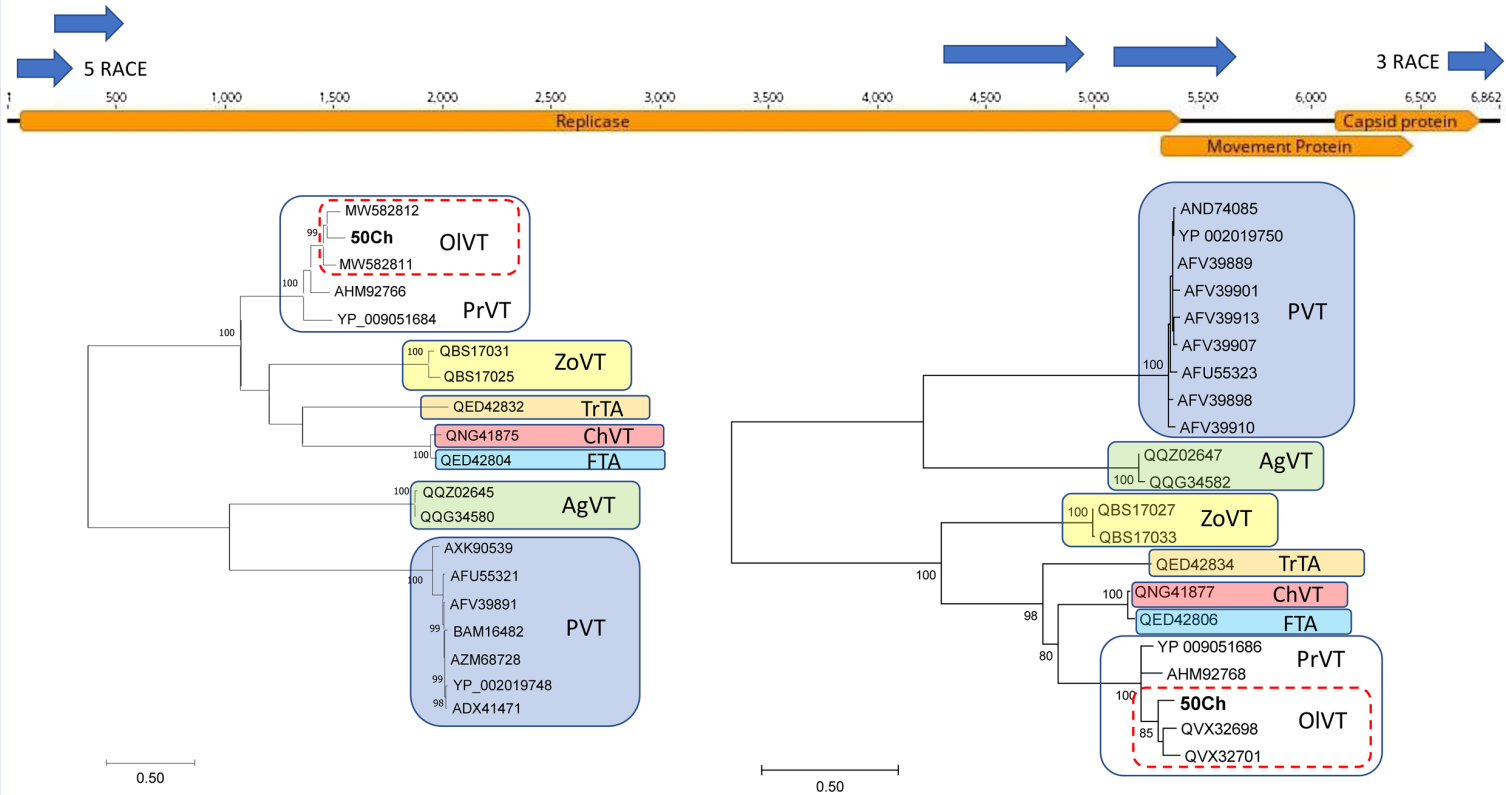
Genome organization of the OlVT 50Ch isolate in 5′–3′ direction. Orange-colored arrows represent open reading frames (ORFs) and dark-colored arrows represent the amplicons used for sequence confirmation. The primers used to generate each amplicon are given in Supplementary Table 1. The aminoacid sequences of REP (left panel) and CP (right panel) were used for the construction of the maximum-likelihood phylogenetic (ML) trees. The ML trees were constructed using MEGA v. 7.1 under the GTT + G + I and GTT + G models of evolution for the REP and CP, respectively. The available viral isolates are reported by their accession numbers shown at the branch tips. The scale bars show the number of substitutions per site, and the bootstrap percentages (1000 re-samples) are indicated on the branches. OlVT: olive virus T, PrVT: prunus Virus T, ZoVT: zostera virus T, TrTA: trichosanthes virus A, ChVT: cherry virus T, FTA: ficus virus A, AgVT: agave virus T, PVT: potato virus T

For sample 50Ch, two contigs (6823 and 5516 nt in length, respectively) exhibited high nt sequence identity (79.59%) to OlVT isolate GR168 (accession no. MW582811) from Greece and 75.68% to prunus virus T (PrVT) isolate C21 (accession no. KF700262) from Italy. The nearly complete betaflexivirus genome of the 50Ch isolate was reconstructed using iterative mapping of the reads to the de novo contigs, with 10,476 reads mapped in total confirming the typical genome organization of a tepovirus (Fig. 2). The close relationship between OlVT and PrVT was also confirmed by the phylogenetic analysis of the REP and CP proteins of 50Ch with those from other tepoviruses (Fig. 2).

### 5′ and 3′ RACE assays and partial confirmation of the new OlVT and OLYaV isolates with Sanger sequencing

The amplicons from REP (365 bps and 523 bps) and MP (352 bps) of OlVT were identical to the sequence obtained with the HTS. The assembly of the reconstructed sequence and RACE products resulted in the 6862-nt-long genomic sequence of the isolate 50Ch (accession no. OK569892), which confirmed the sequence obtained by HTS.

Similarly, for OLYaV, the amplicon from the HSP70h (583bps) shared 100% identity in nucleotides with the OLYaV isolate retrieved by HTS analysis. In addition, the application of 5′ and 3′ RACE assays led to the confirmation of the 5′ end sequence; however, it was not possible to obtain the exact 3′ end despite our efforts using different approaches (different primer pairs, RNA extraction methods etc.).

### OlVT 50Ch isolate sequence comparisons and recombination analysis

**Fig. 3:**
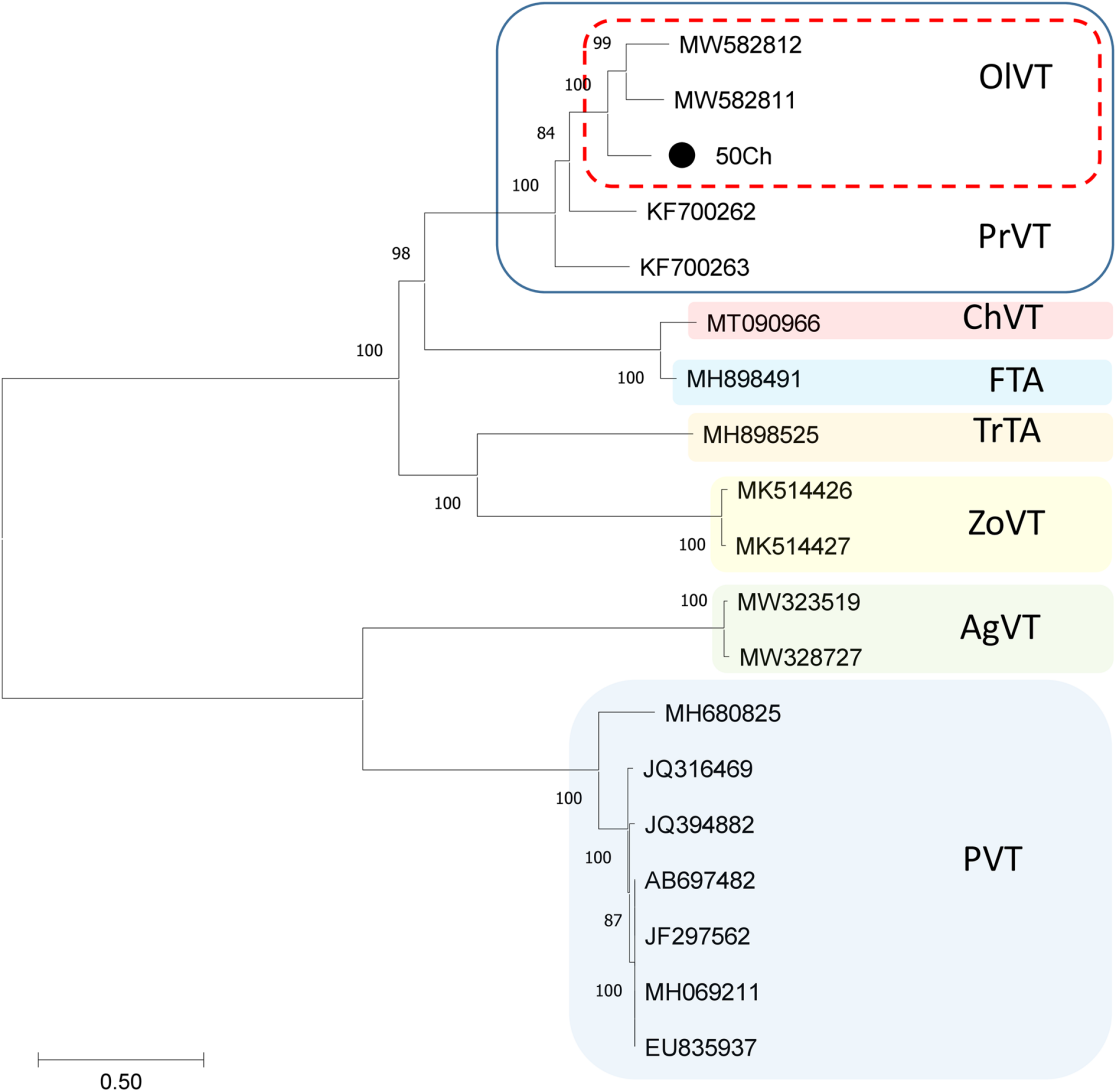
Maximum-likelihood phylogenetic tree constructed by MEGA v 7.1 under the GTT + G + I model of evolution using the full genome from the representative members of the genus *Tepovirus*. The viral sequences are reported by their accession number. The scale bar shows the number of substitutions per site. Bootstrap percentages (1000 re-samples) are indicated on the branches. OlVT: olive virus T, PrVT: prunus virus T, ZoVT: zostera virus T, TrTA: trichosanthes virus A, ChVT: cherry virus T, FTA: ficus virus A, AgVT: agave virus T, PVT: potato virus T. The black cycle indicates the full nucleotide sequence of isolate 50Ch determined in the present study

The 50Ch isolate sequence exhibited 79% nt identity with OlVT GR168 and 74% with PrVT C21 full-length sequences (Suppl. Table 3). The OlVT isolate 50Ch shared 87/87, 80/82, and 92/88% aa identity with the isolates GR168/GR170 in the REP, MP and CP, respectively (Suppl. Table 3). Amino acid identities with the PrVT isolates C21/Aze239 were 82/79, 76/97, and 83/86% in the REP, MP and CP, respectively (Suppl. Table 3). Despite the variability observed with OlVT isolates, 50Ch is classified as a divergent OlVT isolate [13], as also depicted by the phylogenetic analysis of the CP protein (Fig. 2). Yet, a close relation between 50Ch isolate with PrVT is also evident (Fig. 2), and these findings were also supported from the full genome phylogenetic analysis of tepoviruses (Fig. 3).

**Fig. 4:**
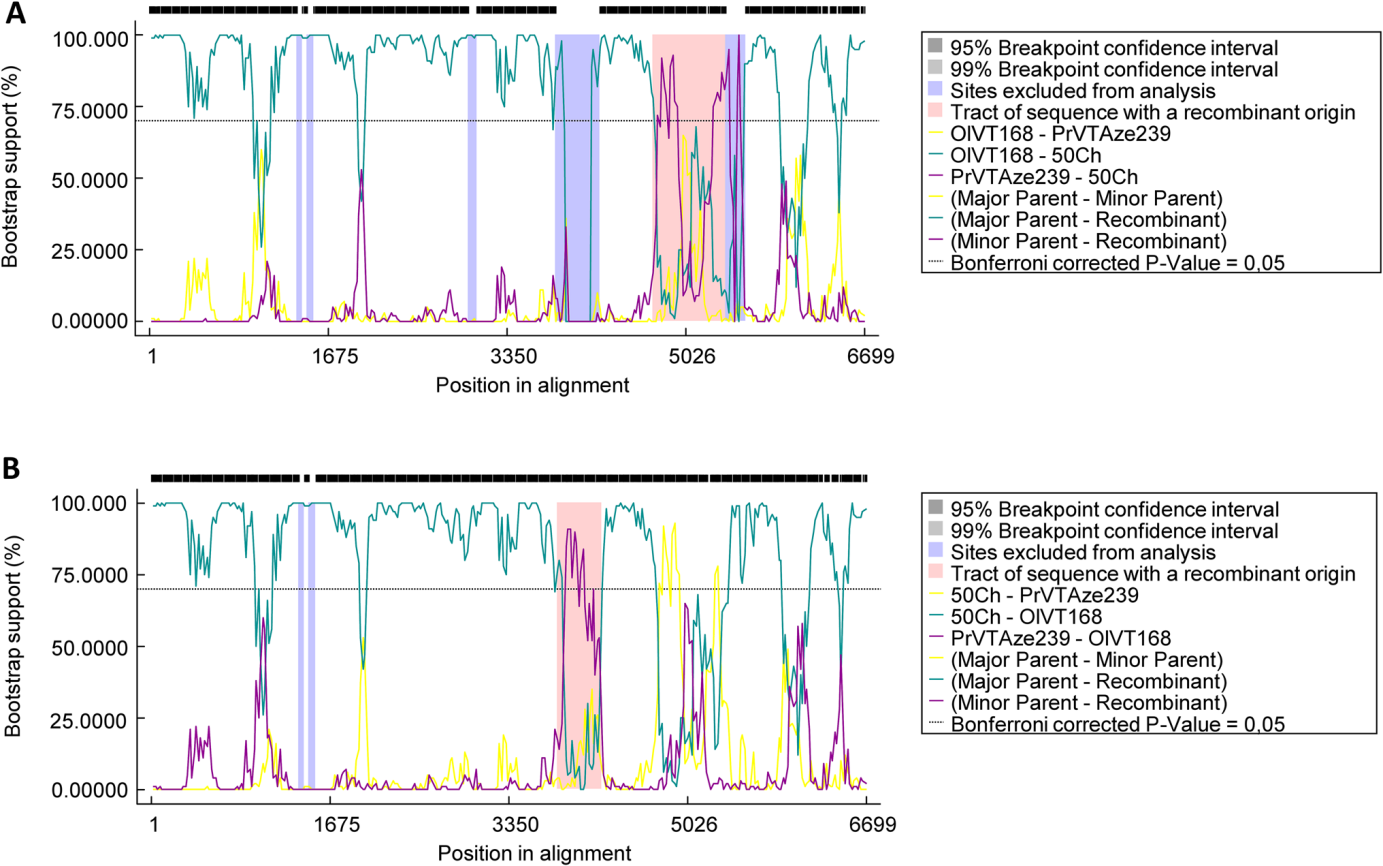
Results of bootscan analysis of recombination events in **A** 50Ch and **B** OlVT-168 genomes using RDP5 software. The analysis was performed using the Jukes and Cantor (1969) model, with a window size of 200 bases, a step size of 20 bases, and a number of bootstrap replicates of 100 as implemented in RDP5 program. Cutoff percentage (70%) is shown by dashed line. The colors of the lines correspond to different pairs of analyzed viruses as indicated in the legend

According to the RDP5 software, 2 putative recombination events were detected with 6 and 5 methods for 50Ch (regions derived from major parent OlVT168: 1-4712, 5400-6699 and region derived from minor parent PrVTze239: 4713–5399) and OlVTGR168 (regions derived from major parent 50Ch: 1-3804, 4220-6699 and region derived from minor parent PrVTze239: 3805-4219), respectively (Fig. 4). However, more tests are needed to identify whether recombination truly occurs between OlVT and PrVT isolates.

### Field surveys and evolutionary relationships of OlVT isolates

Apart from 50Ch, ten more trees were found to be infected with OlVT, thus the prevalence of the virus reached 12.9% of the collected samples (Suppl. Table 2). The comparison of the partial REP and CP nt sequences from four (1Ch, 2Ch, 9Ch and 50Ch, accession no. OK569887, OK569888, OK569889 and OK569892, respectively) and three (1Ch, 2Ch, 50Ch, accession no. OK569890, OK569891, OK569892, respectively) olive isolates (cv. Chondrolia collected from Chalkidiki), respectively, showed high nt intra-host similarities ranging between 88.56 and 97.88% and 92.28–97.43%, respectively, whereas the corresponding sequence similarities with the sequences of OlVT available in database ranged between 83.47–87.29% (REP) and 82.68–86.62% (CP). The similarities of the REP gene with those from PrVT isolates ranged at the same level as those of OlVT (82.2–86.44%), whereas in the CP gene, they were lower (77.87–79.93%).

Phylogenetic trees constructed using the OlVT partial sequences (same procedure described for the tepovirus tree, with zostera virus T as an outgroup) confirmed the results obtained by sequence comparisons. In the CP gene-based tree, the available OlVT isolates grouped in a different clade from the newly identified 1Ch, 2Ch, 9Ch, and 50Ch OlVT isolates, whereas for Rep, based on the bootstrap values, all OlVT isolates are clustered together (Suppl. Figure 1). These results suggest that the currently known OlVT isolates may form two different phylogenetic groups.

### Field surveys and evolutionary relationships of OLYaV isolates

The new nested RT-PCR developed for the specific detection of OLYaV was applied for testing the 85 samples and the virus was found in 20 of them (2 cv. Picual—1 cv. Megaron—2 cv. Kalamon—1 cv. Chondrolia—14 cv. Arbequina) (Suppl. Table 2). Partial OLYaV HSP70h nt sequences were determined from two isolates (EL22, cv. Arbequina and 2Ch, cv. Chondrolia) collected from Thessaloniki and Chalkidiki, respectively. Isolate EL22 (accession no. OK569884) was 97.97% similar at nt level to isolate 26Arbequina (HQ316959) from Spain (the nt similarities compared to the full sequenced isolates V64 from Spain and CS1 from Brazil were 95% and 72%, respectively), whereas isolate 2Ch (accession no. OK569885) showed 99.08% nt identities to the Greek isolate OLYaV-Gr/Ms (accession no. LR593885) (nt similarities with the full sequenced isolates V64 from Spain and CS1 from Brazil were 96.5% and 72%, respectively). The similarities among EL22 και 2Ch were 93.98% and with isolate OL2 they were 94.19% and 99.83%, respectively, at nt level (data not shown).

The phylogenetic tree constructed using 350 bps of the available OLYaV HSP70h partial sequences (most isolates deposited in the database were of that size and for that reason the homologous Greek sequences were trimmed to fit) confirmed the close relationship of the Greek isolates. All three isolates determined in the present study clustered in the same clade with isolates OLYaV-Gr/Ms and 8Gaidourelia from Greece and several others, most of which were isolated from countries of the Mediterranean basin. A small subgroup consisted only of Greek isolates was also observed (isolates OL2, Ch2, and OLYaV-Gr/Ms) (Suppl. Figure 2).

## Discussion

The presence of OLYaV has been reported recently in Greece [4, 5]. It was also reported from the National Clonal Germplasm Repository collection of the USA in olives cvs Gaidourelia and Conservolia originating from Greece [18]. However, only small parts of the HSP70h sequence were so far available from all isolates. In this study, the first complete sequence of a Greek OLYaV isolate has been determined. Since there are only two full OLYaV sequences reported so far, V64 from Spain and CS1 from Brazil, this finding increases our knowledge on the genetic diversity of the virus and will further facilitate its classification as a member of a new genus within the family *Closteroviridae* [11, 12].

In our study, OLYaV was frequent (23.5%) in the tested samples similarly to reports from Lebanon, Italy, Spain, and Tunisia [12, 26–29]. On the contrary, in previous surveys conducted in Greece, both in olive orchards [5] and in a germplasm collection of the Institute of Olive Tree, Subtropical Crops and Viticulture (IOSV) in Chania [4], its incidence was very low (1.2 and 5%, respectively) and similar to that reported in Albania (2%) from Luigi et al. [30].

An explanation provided in the previous studies, regarding the low infection rate of OLYaV in Greece, was related to the absence or low populations of the putative psyllid vector (*Euphyllura olivinae*) in Greek olive orchards [4, 5]. However, from our study, we conclude that high genetic variability and/or low virus titer, both frequently encountered in OLYaV and in other closteroviruses, could also explain the differences observed [12, 28, 29].

The full genome of the Greek isolate OL2 determined herein shares high similarity with the newly described isolate V64 from Spain. Both OL2 and V64 are divergent from the CS1 from Brazil, the first available full sequence. The similarity in nt of the HSP70h region [15] used so far for primers design for the detection of OLYaV was approximately 80% suggesting that this region is highly divergent and therefore cannot be reliable for virus detection [12]. Although several OLYaV HSP70h partial sequences were determined using the above-mentioned specific primers, the high divergence observed in this gene could affect the specificity of the methods used broadly so far in certification schemes as isolates with higher variability could leak detection, similarly to other closteroviruses [31]. This hypothesis is also supported by the sequence analysis comparison of the Greek OLYaV isolates determined in the present study with the homologous sequences from 123 virus isolates available from the database (data not shown) which has shown a nt identity in the partial HSP70h gene ranging from 68 to 100%.

Recently, two new protocols have been reported for the specific detection of OLYaV. The RT-qPCR method developed by Campos et al. [28] using primers targeting in HSP90h along with double-stranded RNA (dsRNA) as template has resulted in the detection of a higher number of OLYaV-infected trees when compared to the conventional RT-PCR assay where total RNA extract was used. Also, the application of a new RT-PCR assay developed by Ruiz-Garcia et al. using primers targeting the highly conserved 5′UTR of the virus revealed a 43.5% incidence of OLYaV in Spanish olive groves [12]. The nested RT-PCR method developed and applied in the present study, even though it targeted the HSP70h, exhibited high sensitivity due to the implementation of the extra PCR step and the use of degenerate primers and was able to successfully detect the virus in 20 out of 85 samples showing infection rates similar to those observed with the new methods described above. Therefore, it could be applied for the reliable detection of OLYaV in olive trees.

OlVT is a newly described olive virus that has been so far reported only in Greece [5]. In our study, a divergent virus genotype, which seems to be widespread in Northern Greece, was identified thus highlighting the presence of high genetic diversity within OlVT. However, more data are needed to further characterize genome diversity within the species, given that only three full genomic sequences of OlVT are currently available: GR170 and GR168 from Central Greece (Fthiotida) [5] and the 50Ch isolate from Northern Greece reported herein.

Ten trees were found infected with OlVT corresponding to 12.9% of the olive trees tested, whereas Xylogianni et al. [5] reported a lower OlVT infection rate of 4.4%. The discrepancy observed could be due to the application of different detection protocols and/or the different geographical regions sampled in Greece. Herein, olive samples originated from Northern Greece and especially Chalkidiki and Thessaloniki, whereas Xylogianni et al. [5] tested samples from Central and Southern Greece.

Comparison of the partial REP sequence of four OlVT isolates and partial CP sequence of three isolates showed high similarities (88.56–97.88% in REP and 92.28–97.43% in CP), whereas the respective sequence similarities with the isolates available in database were slightly lower for REP (83.47–87.29%) and even lower for CP (82.68–86.62%). Nucleotide similarity ranging between 75.6 and 99.3% was previously recorded in the partial MP_CP region from Xylogianni et al. [5].

High genetic diversity has been previously identified also for the closely phylogenetically related PrVT. The first reported full sequences of PrVT isolates were obtained from *Prunus* trees, C21 (sweet cherry), and Aze239 (plum) and shared 78.7% aa identity in the MP (80.1% nt identity), 79.9% in the REP (72% nt identity), and 89.5% in the CP (80.3% nt identity) [32].

It is well known that recombination in RNA viruses is a powerful driving force for generating new variants [33–35]. Previous analyses indicated frequent recombination events between several members of the *Betaflexiviridae* family [36, 37]. Thus, it is possible that RNA recombination also plays a role in OlVT variation and evolution. In the present study, two putative recombination events were observed between OlVT isolates 50Ch and Gr168 with PrVTAze239 indicating that OlVT might have derived from PrVT. Yet, a large collection of OlVT and PrVT isolates and further analysis are necessary to verify if recombination naturally exists between the two viruses and to generate more detailed information about their population structure and origin of the species.

In conclusion, this is the first report of the almost complete genome of a Greek OLYaV isolate and the complete genome characterization of a divergent isolate of OlVT. Especially for OlVT, it is yet unknown how this virus affects olive trees and if it is the causal agent of any symptoms and disease. Therefore, additional studies are necessary to evaluate the presence and impact of olive viruses in Greece and worldwide. The improved detection methods developed in this study will aid toward this direction.

## Supplementary Information

Below is the link to the electronic supplementary material.

Supplementary file1 (TIFF 1269 kb) Suppl. Fig. 1. Maximum-likelihood phylogenetic tree constructed by MEGA v 7.1 under the T92+G and K2+G models of evolution using the partial Replicase and CP genome sequences respectively, from prunus virus T and olive virus T isolates as well as the Greek isolates determined in the present study, which are indicated by black circle. The viral sequences are reported by their accession number. The scale bar shows the number of substitutions per site. Bootstrap percentages (1000 re-samples) are indicated on the branches. ZoVT (zostera virus T) was used as outgroup to root the tree

Supplementary file2 (TIFF 978 kb) Suppl. Fig. 2. Maximum-likelihood phylogenetic tree using the GTR+I substitution model conducted by MEGA X of the partial HSP70 genome sequences (of 350 bp) from all OLYaV isolates available in database and the Greek isolates determined in the present study. Viral sequences are reported by their accession number. The scale bar shows the number of substitutions per site. Bootstrap percentages (1000 re-samples) are indicated on the branches. The Greek isolates determined in this study are indicated by black circle, whereas the other three available Greek isolates are indicated by black triangle

Supplementary file3 (XLSX 23 kb)

## Data Availability

The nucleotide sequences reported here have been deposited in the GenBank database under accession numbers: OK569884, OK569885, OK569886, OK569892, OK569887, OK569888, OK569889, OK569890, OK569891.
