# Supplementary figures and images for: Identification and complete genome sequencing of a divergent olive virus T isolate and an olive leaf yellowing-associated virus isolate naturally infecting olive trees in Greece
Source: Virus Genes. 2022 Sep 24;58(6):560–9. doi: 10.1007/s11262-022-01934-4 (PMC9636108; doi:10.1007/s11262-022-01934-4)

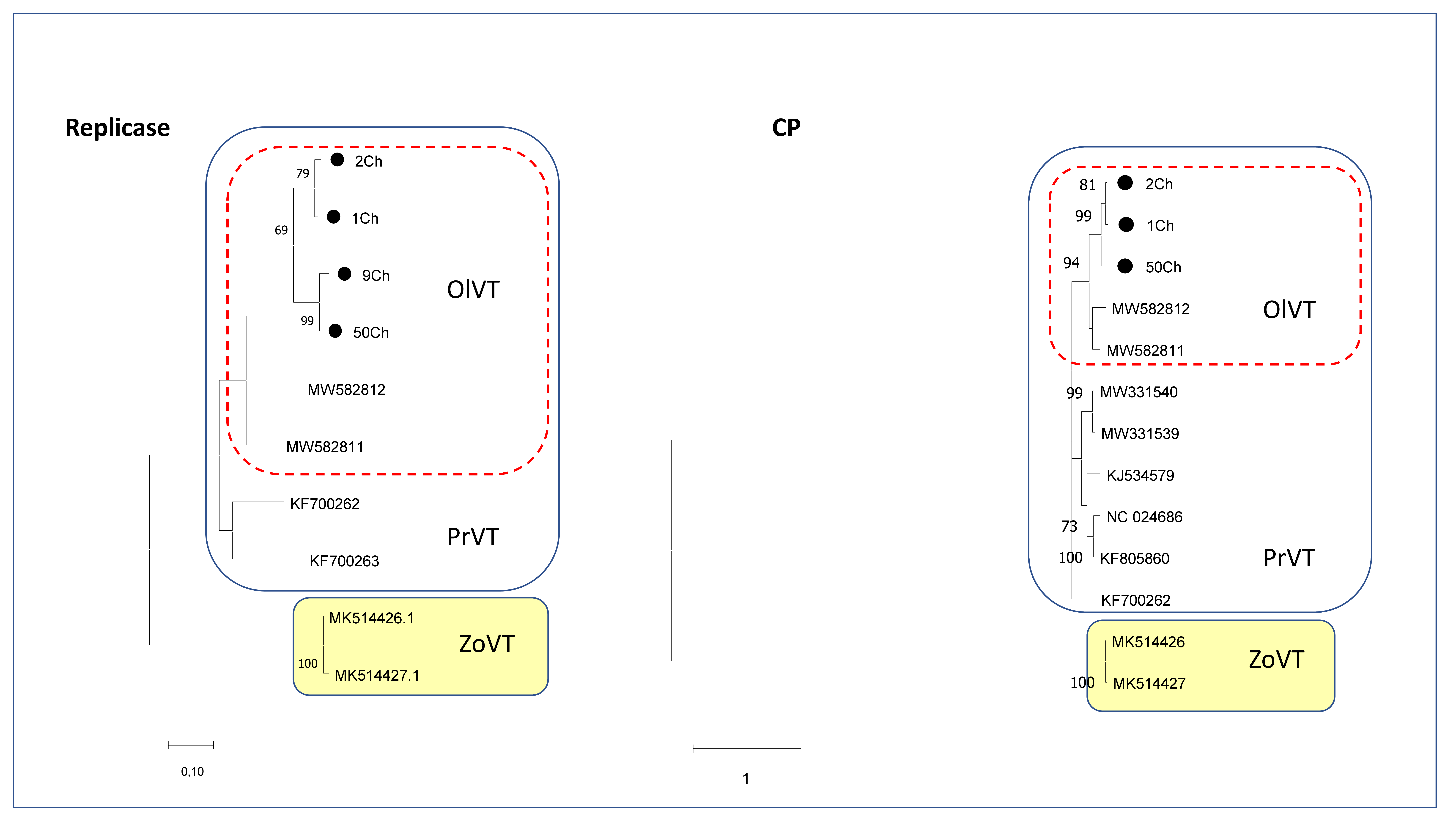

Supplement: Supplementary file 1 — Supplementary file1 (TIFF 1269 kb) Suppl. Fig. 1. Maximum-likelihood phylogenetic tree constructed by MEGA v 7.1 under the T92+G and K2+G models of evolution using the partial Replicase and CP genome sequences respectively, from prunus virus T and olive virus T isolates as well as the Greek isolates determined in the present study, which are indicated by black circle. The viral sequences are reported by their accession number. The scale bar shows the number of substitutions per site. Bootstrap percentages (1000 re-samples) are indicated on the branches. ZoVT (zostera virus T) was used as outgroup to root the tree [file 11262_2022_1934_MOESM1_ESM.tiff]
